# Do primary and specialized care physicians know and use coordination mechanisms?

**DOI:** 10.11606/s1518-8787.2020054002475

**Published:** 2020-11-17

**Authors:** Lívia dos Santos Mendes, Patty Fidelis de Almeida

**Affiliations:** I Instituto Multidisciplinar Vitória da ConquistaBA Brasil Universidade Federal da Bahia. Instituto Multidisciplinar em Saúde. Pós-Graduação em Saúde Coletiva. Vitória da Conquista, BA, Brasil; II Universidade Federal Fluminense NiteróiRJ Brasil Universidade Federal Fluminense. Instituto de Saúde Coletiva. Niterói, RJ, Brasil

**Keywords:** Intersectoral Collaboration, Comprehensive Health Care, Health Care Levels, Health Services Administration

## Abstract

**OBJECTIVES:**

To analyze if primary and specialized care physicians know and use care coordination mechanisms between healthcare levels.

**METHODS:**

Cross-sectional survey study, with the application of the COORDENA-BR instrument to primary and specialized care physicians in a public heathcare network, medium-sized municipality, from June to October 2019. The questionnaire addresses knowledge, frequency of sending and receiving, purpose, characteristics and difficulties in using feedback or mutual adaptation and standardization mechanisms to promote coordination of care service between healthcare levels.

**RESULTS:**

Feedback instruments such as referral and reply letters, hospital discharge report and WhatsApp are widely known by professionals of both levels, without significant differences. Clinical sessions and protocols are not well-known, especially in specialized care, which supposes a low usage of standardization mechanisms to a better coordination between the healthcare levels. Despite being well-known and easy, traditional feedback instruments such as referral and reply letters are not widely used. Fewer physicians knew the protocols, mainly in specialized care. They pointed difficulties in their application, such as insufficient exams and unavailable supplies in the healthcare network. Clinical sessions were unknown and registered low participation frequency. Care overload, low institutionalization and time constraints were barriers identified for the incorporation of care coordination mechanisms in the work process in primary and specialized care, in addition to those related to the provision of health services in the network.

**CONCLUSION:**

We conclude the fragmentation of the system and care can be faced in the complementarity of measures that make it possible to know the mechanisms, develop professional skills, institutionalize and promote organizational conditions for the effective use of coordination mechanisms throughout the healthcare network.

## INTRODUCTION

The diversity and complexity of health needs in the face of nutritional, epidemiological and demographic transition combined with technological advances incorporated into heath care have, somehow, expanded the available therapeutic arsenal and configured a pattern of multiple contacts with health professionals and services, especially in cases of multimorbidity^1^^,^^2^, expanding the need for strategies for the care coordination. The use of instruments for communication and care articulation between these healthcare levels strengthens the coherence, efficiency and quality of care^3^. Despite the consensus around their need, a set of evidences indicates incipience in the knowledge and use of coordination mechanisms by professionals in different contexts of health systems^4^^,^^5^.

The duplication of diagnostic and therapeutic procedures, unnecessary referrals and the non-conciliation of treatments are both results and one of the facets that show the incoherence between the healthcare levels and their low quality^6^. The lack of interaction and trust between professionals in primary health care (PHC) and specialized care (SC) are part of the problem^7^^,^^8^, whose coping cannot depend only on the intention and initiative of health professionals, being dependent on labor, organizational, relational conditions and the characteristics of the system as a whole^9^.

Coordination instruments aim to minimize the barriers between the healthcare levels, favoring continuity by transfer of information, exchange of knowledge and improvement of care^10^^,^^11^. Coordination mechanisms can be classified as standardization mechanisms and mutual feedback or adaptation mechanisms^11^^,^^12^. Standardization mechanisms aim to harmonize or systematize the work process, professionals' skills and work results, such as clinical sessions and shared protocols. Mutual adaptation or feedback mechanisms are based on communication and the formal and informal information exchange between professionals for shared care planning, such as referral forms and reply letters, discharge report and phone line^12^. These instruments are important for promoting adjustments in clinical situations that involve a large set of information, highly specialized and, at the same time, interdependent activities^13^.

The proper use of coordination mechanisms would be one of the facilitators of communication and integration between PHC and SC^4^^,^^14^. National studies on the topic often focus on PHC teams and physicians^14^^,^^15^, since the guidelines of national policies define that the care coordination in health care networks (HCN) is responsibility of this healthcare level. Based on the experience and perception of the two main actors involved in the activities of articulation of care between the different services of the HCN, this article aims at analyzing if physicians from PHC and SC know and use mechanisms to care coordination between healthcare levels.

## METHODS

This is a cross-sectional survey study conducted in PHC and SC services in the Unified Health System (SUS) network of a medium-sized municipality (338,480 inhabitants in 2019) in the state of Bahia, Brazil. A census of PHC and SC medical professionals working in these services was conducted between June and October 2019. The PHC had coverage of 60%, 47% by the Family Health Strategy (FHS) and 13% by traditional health centers (HC)^16^.

The sample included all PHC doctors (FHS and traditional HC) and medical specialists that received regular primary care referrals for specialized consultations. Participants worked for at least three months in the respective service at the beginning of the field research, according to information from the municipal administration. We interviewed 120 of the 136 physicians. Individuals interviewed and losses are described according to their service in Table 1.

**Table 1:** Physicians of primary health care and specialized care interviewed according to health service care. Medium-sized municipality, Northeast, Brazil, 2019.

Type of service			Number of physicians	Number of losses	Number of respondents
PHC	FHS	Countryside	16	2	14
		Urban area	28	2	26
	**Total of FHS physicians**		**44**	**4**	**40**
	**Total of BHC physicians**		**26**	**2**	**24**
**Total of PHC physicians**			**70**	**6**	**64**
SC	Specialized medical center		52	9	43
	Mental health outpatient clinic		5	-	5
	Rehabilitation clinic		2	-	2
	BHC		7	1	6
**Total of specialists**			**66**	**10**	**56**
**Total**			**136**	**16**	**120**

PHC: primary health care; SC: specialized care; FHS: Family Health Strategy; BHU: Basic Health Units.

To conduct our study, the validated and translated instrument into Portuguese COORDENA-BR^a^, which is based on the theoretical model developed by Vázquez et al.^2^ and Vargas et al.^9^, was used to analyze the coordination of care between healthcare levels. The complete questionnaire addresses: experience in coordinating information and in clinic management between healthcare levels and general perception about coordination between levels in the network; professionals' interaction factors; coordination mechanism knowledge and use; suggestions for improvement; organizational, work factors and attitudes related to coordination between levels; sociodemographic data of the interviewees. The items related to the knowledge and use of coordination mechanisms between levels are the focus of our article.

The questionnaire addresses knowledge, frequency of sending and receiving, purpose, characteristics and difficulties in using feedback (or mutual adaptation) and standardization mechanisms to promote coordination of care between healthcare levels, which comprised the analyzed variables. The questionnaire underwent minor adjustments to the local scenario (for example, the type of protocol adopted in the HCN) and some updates, such as the addition of a question about knowledge on the electronic medical record and Telehealth implemented in the municipality, according to management information, and WhatsApp, technology increasingly used in interprofessional communication^17^. Use purposes and difficulties were identified by open questions. Likewise, there were open fields to explain the reasons for not regularly receiving the referral and reply letters and the discharge report.

For closed questions, the Likert scale and dichotomous answers (yes / no) were used, in addition to multiple choice questions. Some variables of interest were categorized for a better comparison between PHC and SC physicians. The answers “always” and “often” as well as “daily” and “weekly” were considered as high frequency; “Sometimes” and “very few times” as well as “monthly” and “less frequently” as low frequency; and “never” represented the non-use of the instrument (zero frequency). The questionnaire was digitized using the KoBo Toolbox 1.4.8 software, available on Samsung Galaxy Tab A tablets.

Data was collected by face-to-face interviews conducted by trained researchers, with an average duration of 26.6 minutes, being recorded on an audio device for transcription and categorization of open questions. To guarantee the quality of collection and reliability of data, field activities were directly monitored and questionnaire completion in the database were evaluated.

A descriptive analysis of the variables was performed by healthcare level, using absolute (n) and relative (%) frequencies. Data were processed using the Stata program, version 15.0 (Stata Corporation, College Station, USA). Statistical differences between the proportions were evaluated by Pearson's chi-squared test. Qualitative data from open responses were categorized.

This study was approved by the Research Ethics Committee of the Universidade Federal da Bahia, under opinion no. 3.334.464 and CAAE: 09503419.1.0000.5556 with permission of the municipality.

## RESULTS

### Sample Characteristics

The largest proportion of professionals was of men (57%), with women mostly in PHC (55%), younger, aged 25 to 34 years (40%). SC physicians were professionals for a longer time and most of them (55%) attended public universities. In PHC, most of the professionals attended private colleges and about 60% had no medical residency training. Most of the specialists had employment contracts with a lower week workload (80%) and also worked in the private sector (93%), unlike PHC professionals (Table 2).

**Table 2:** Sample characterization. Medium-sized municipality, Northeast, Brazil, 2019.

Characteristics		PHC (n = 64) n (%)	SC (n = 56) n (%)	Total (n = 120) n (%)
Gender				
	Female	35 (54.7)	17 (30.4)	52 (43.3)
	Male	29 (45.3)	39 (69.6)	68 (56.7)
Age (years)				
	25–34	26 (40.6)	11 (19.6)	37 (30.8)
	35–49	22 (34.4)	34 (60.7)	56 (46.7)
	> 50	16 (25.0)	11 (19.6)	27 (22.5)
Nationality				
	Brazilian	64 (100.0)	56 (100.0)	120 (100.0)
Training time (years)				
	≤ 2	15 (23.4)	0 (0.0)	15 (12.5)
	3–10	26 (40.6)	15 (26.8)	41 (34.2)
	11–20	7 (10.9)	24 (42.8)	31 (25.8)
	> 20	16 (25.0)	17 (30.4)	33 (27.5)
Education institution				
	Public	24 (37.5)	31 (55.4)	55 (45.8)
	Private	40 (62.5)	25 (44.6)	65 (54.2)
Medical specialization				
	No Medical Residency	38 (59.4)	1 (1.8)	39 (32.5)
	Medical clinic	9 (14.1)	0 (0.0)	9 (7.0)
	Pediatrics	7 (10.9)	5 (8.9)	12 (10.0)
	Obstetrics and gynecology	3 (4.7)	6 (10.7)	9 (7.0)
	Others	7 (10.9)	44 (78.6)	51 (42.5)
Working hours per week				
	20–32	42 (65.6)	45 (80.4)	87 (72.5)
	≥ 40	22 (34.4)	11 (19.6)	33 (27.5)
Works in the private sector				
	Yes	31 (48.4)	52 (92.9)	83 (69.2)
	No	33 (51.6)	4 (7.1)	37 (30.8)

PHC: primary health care; SC: specialized care.

### Knowledge and Use of Care Coordination Mechanisms between Healthcare Levels

The formal mechanism of coordination between levels best known among PHC physicians was the referral form and reply letters (86%), followed by the discharge report (78%). Specialists knew more about the discharge report (91%), followed by the referral and reply letters (70%). Regarding standardization instruments, the protocols shared between levels, developed by the Brazilian Ministry of Health and the municipality, were better known by PHC physicians when compared with specialists (p = 0.004). Table 3 shows that the PHC and SC joint clinical sessions were little known by physicians of both levels, and practically unknown by specialists (p = 0.001).

**Table 3:** Existence and frequency of use of coordination mechanisms between healthcare levels. Medium-sized municipality, Northeast, Brazil, 2019.

		PHC (n = 64) n (%)	SC (n = 56) n (%)	Total (n = 120) n (%)	p
Existence of the instrument					
Referral form and reply letters					
	Yes	55 (85.9)	41 (73.2)	96 (80.0)	0.082
	No	9 (14.1)	15 (26.8)	24 (20.0)	
Hospital discharge report					
	Yes	50 (78.1)	51 (91.1)	101 (84.2)	0.053
	No	14 (21.9)	5 (8.9)	19 (15.8)	
MH and municipal protocols shared between levels					
	Yes	42 (65.6)	22 (39.3)	64 (53.3)	**0.004**
	No	22 (34.4)	34 (60.7)	56 (46.7)	
Joint clinical sessions between PHC and SC physicians					
	Yes	29 (45.3)	9 (16.1)	38 (31.7)	**0.001**
	No	35 (54.7)	47 (83.9)	82 (68.3)	
Institutional phone line					
	Yes	41 (64.1)	51 (91.1)	92 (76.7)	**< 0.001**
	No	23 (35.9)	5 (8.9)	28 (23.3)	
Telehealth					
	Yes	35 (54.7)	5 (8.9)	40 (33.3)	**< 0.001**
	No	29 (45.3)	51 (91.1)	80 (66.7)	
Electronic medical record					
	Yes	29 (45.3)	6 (10.7)	35 (29.2)	**< 0.001**
	No	35 (54.7)	50 (89.3)	85 (70.8)	
WhatsApp:					
	Yes	56 (87.5)	54 (96.4)	110 (91.7)	0.102
	No	8 (12.5)	2 (3.6)	10 (8.3)	
Frequency^a^					
Referral form and reply letters sending^b^		n = 55	n = 41	n = 96	**0.038**
	High	24 (43.6)	8 (19.5)	32 (33.3)	
	Low	22 (40.0)	21 (51.2)	43 (44.8)	
	Never	9 (16.4)	12 (29.3)	21 (21.9)	
Referral form and reply letters receiving^c^		n = 55	n = 41	n = 96	**0.012**
	High	2 (3.6)	7 (17.1)	9 (9.4)	
	Low	26 (47.3)	24 (58.5)	50 (52.1)	
	Never	27 (49.1)	10 (24.4)	37 (38.5)	
Hospital discharge report receiving		n = 50	n = 51	n = 101	-
	High	22 (44.0)	-	-	
	Low	25 (50.0)	-	-	
	Never	3 (6.0)	-	-	
Use of MH and municipal protocols shared between levels		n = 42	n = 22	n = 64	**0.006**
	High	39 (92.8)	14 (63.6)	53 (82.9)	
	Low	2 (4.8)	2 (9.1)	4 (6.2)	
	Never	1 (2.4)	6 (27.3)	7 (10.9)	
Participation joint clinical sessions between PHC and SC physicians		n = 29	n = 9	n = 38	**0.002**
	High	19 (65.5)	1 (11.1)	20 (52.6)	
	Low	10 (34.5)	6 (66.7)	16 (42.1)	
	Never	0 (0.0)	2 (22.2)	2 (5.3)	
WhatsApp Use		n = 56	n = 54	n = 110	0.259
	High	7 (12.5)	2 (3.7)	9 (8.2)	
	Low	22 (39.3)	22(40.7)	44 (40.0)	
	Never	27 (48.2)	30 (55.6)	57 (51.8)	

PHC: primary health care; SC: specialized care; MH: Ministry of Health.

a Questions answered only by professionals that knew about the existence of the instrument.

b The referral form sending was answered by the PHC physicians and the reply letters form sending by the SC physicians.

b The referral form receiving was answered by the PHC physicians and the reply letters form sending by the SC physicians.

Values with statistical significance are shown in bold.

About 36% of PHC physicians were unaware of the existence of an institutional phone line and 45% were unaware of Telehealth. Among SC physicians, the phone line was widely known, and Telehealth was unknown. The electronic medical record seemed to be present in part of the PHC services, but absent in the SC, since it was unknown by most of the professionals. WhatsApp was the most popular instrument by professionals at both levels. We observed a significant difference between the knowledge of PHC and SC physicians about most of the coordination mechanisms of care service (Table 3).

After recognizing the existence, we sought to understand the frequency of use of the coordination mechanisms, categorized as “high,” “low” or “never.” Most professionals knew the referral and reply letters instruments. However, only 44% of PHC physicians mentioned high sending frequency of the referral form to the specialist, who, in turn, mentioned low receiving frequency. On the other hand, fewer specialists knew such instruments, and few frequently sent reply letters to PHC physicians, about half of whom reported never receiving them. Discharge report receiving by physicians at the first level was more frequent. The fact that these instruments are sent via patient was mentioned by the interviewees as a reason for not receiving them. Among the PHC physicians that knew the shared protocols (42), the most had a high frequency of using this tool (93%). Few specialists knew it (22); of these, 64% used it with high frequency. The clinical sessions were little known by professionals of both levels and even less used by specialists. WhatsApp was widely known by professionals (more frequent in SC services), but with little or no use (Table 3).

### Purpose and Characteristics of the Use of Coordination Mechanisms between Healthcare Levels

Among the professionals that knew the mechanisms and mentioned using them with some frequency, their purpose was evaluated by open questions, whose content was categorized. They mostly considered that the referral, reply letters and discharge forms were used to exchange information between healthcare levels. For PHC and SC physicians, the main function of the protocols was to guide the care and standardize the service, with quality of care and reduction of costs being mentioned. In the opinion of the few physicians that knew the clinical sessions, the main objectives would be to improve knowledge and standardize conducts (Table 4).

**Table 4:** Purpose and characteristics of the use of coordination mechanisms between healthcare levels. Medium-size municipality, Northeast, Brazil, 2019.

			PHC n (%)	SC n (%)	Total n (%)
Purpose^a^ ^b^					
Referral form and reply letters			n = 46	n = 29	n = 75
	Information exchange		38 (82.6)	28 (96.5)	66 (88.0)
	Patient referral		10 (21.7)	1 (3.4)	11 (14.7)
	PHC monitoring		2 (4.3)	9 (31.0)	11 (14.7)
	Streamline access to the specialist		4 (8.7)	0 (0.0)	4 (5.3)
	Bureaucracy		2 (4.3)	1 (3.4)	3 (4.0)
Hospital discharge report			n = 47	-	-
	Information exchange		35 (74.5)	-	-
	PHC monitoring		17 (36.2)	-	-
Shared protocols			n = 41	n = 16	n = 57
	Service guidance		19 (46.3)	9 (56.2)	28 (49.1)
	Care standardization		19 (46.3)	7 (43.7)	26 (45.6)
	Care quality		6 (14.6)	1 (6.2)	7 (12.3)
	Cost reduction		1 (2.4)	2 (12.5)	3 (5.3)
	Articulation favoring		3 (7.3)	0 (0.0)	3 (5.3)
	Unnecessary referral prevention		3 (7.3)	0 (0.0)	3 (5.3)
	Bureaucracy		1 (2.4)	0 (0.0)	1 (1.7)
Clinical sessions			n = 29	n = 7	n = 36
	Improve knowledge		17 (58.6)	3 (42.8)	20 (55.5)
	Conduct unification		13 (44.8)	4 (57.1)	17 (47.2)
	Case discussion		5 (17.2)	4 (57.1)	9 (25.0)
	Experience exchange		3 (10.3)	3 (42.8)	6 (16.7)
WhatsApp			n = 29	n = 24	n = 53
	Ease/speed		17 (58.6)	21 (87.5)	38 (71.7)
	Information exchange		11 (37.9)	13 (54.2)	24 (45.3)
	Streamline access to the specialist		6 (20.7)	1 (4.2)	7 (13.2)
Use characteristics					
Referral form and reply letters^a^					
	Referral form and reply letters sending method		n = 46	n = 29	n = 75
		Via patient	37 (80.4)	28 (96.6)	65 (86.7)
		Via regulation center	9 (19.6)	1 (3.4)	10 (13.3)
	Referral and reply letters form receiving method		n = 28	n = 31	n = 59
		Via patient	27 (96.4)	29 (93.5)	56 (94.9)
		Via regulation center	1 (3.6)	2 (6.5)	3 (5.1)
	Information contained in the referral^c^		-	n = 31	-
		Treatments	-	20 (64.5)	-
		Diagnosis	-	20 (64.5)	-
		Personal information	-	29 (93.5)	-
		Clinical history	-	24 (77.4)	-
		Exams	-	18 (58.0)	-
		Reason for referral	-	31 (100.0)	-
	Information contained in the reply letters^c^		n = 28	-	-
		Treatments	25 (89.3)	-	-
		Diagnosis	26 (92.9)	-	-
		Personal information	19 (67.9)	-	-
		Clinical history	18 (64.3)	-	-
		Exams	17 (60.7)	-	-
	Reply letters attends the referral reason.		n = 28	-	-
		High	16 (57.1)	-	-
		Low	10 (35.7)	-	-
		Never	2 (7.2)	-	-
	The reply letters is received in a timely manner to make decisions.		n = 28	-	-
		High	9 (32.1)	-	-
		Low	15 (53.6)	-	-
		Never	4 (14.3)	-	-
Hospital discharge report^a^					
	Receive information in a timely manner to make decisions.		n = 47	-	-
		High	16 (34.0)	-	-
		Low	29 (61.7)	-	-
		Never	2 (4.3)	-	-
	Hospital discharge report receiving method		n = 47	-	-
		Via patient	47 (100.0)	-	-
	Information received in the hospital discharge report^c^		n = 47	-	-
		Pharmacological treatment	47 (100.0)	-	-
		Diagnosis	47 (100.0)	-	-
		Reason of hospitalization	46 (97.9)	-	-
		Procedure performed	47 (100.0)	-	-
		Exam results	40 (85.1)	-	-
		Follow-up indications	30 (63.8)	-	-
Shared protocols^d^					
Participates in training on the use of shared protocols			n = 42	n = 22	n = 64
	High		27 (64.3)	8 (36.4)	35 (54.7)
	Low		15 (35.7)	14 (63.6)	29 (45.3)
	Never		0 (0.0)	0 (0.0)	0 (0.0)
Clinical sessions^d^					
The institution provides time to participate in clinical sessions			n = 29	n = 9	n = 38
	Yes		20 (69.0)	4 (44.5)	24 (63.2)
	Sometimes		6 (20.7)	2 (22.2)	8 (21.0)
	No		3 (10.3)	1 (11.1)	4 (10.5)
	My area of expertise is not included		0 (0.0)	2 (22.2)	2 (5.3)
	Content		n = 29	n = 7	n = 36
		Adequate	27 (93.1)	7 (100.0)	34 (94.4)

PHC: primary health care; SC: specialized care.

a n defined by the number of professionals that presented some frequency of using or receiving the instrument, according to Table 3.

b Categorized open questions.

c Multiple choice questions that admitted more than one answer.

d n defined by the number of professionals that knew the existence of the instrument, according to Table 3.

The use of the phone line and WhatsApp was justified by the ease and speed, although most did not use them (Table 3). Many PHC physicians reported that this communication tool could speed up access to the specialist in situations of greatest urgency, in addition to ensuring that the information reached the other level (Table 4), which did not happen in the case of the referral and reply letters conducted by the user. Qualitative data showed that physicians used phone and WhatsApp to contact only professionals they already knew. PHC physicians that worked in units in rural areas stressed that access to the internet was made possible by the professional himself.

Regarding the characteristics of the use of the coordination mechanisms, in the referral form filled in by the PHC physicians, the specialists received more information about the reasons for referral (100%), personal data and clinical history. In the reply letters by specialists, physicians at the first level received more information about treatments and diagnosis (Table 4). Exam results were the least frequent information. Among the PHC physicians that received the reply letters (28), half considered that they answered the reason for the referral and only one third arrived in a timely manner. Nevertheless, more than half of the specialists did not know or did not use such instruments.

According to most PHC physicians, hospital discharge reports were also not received in a timely manner for decision-making. All professionals considered that this instrument contained information on pharmacological treatment, diagnosis and procedures performed. However, the indications for monitoring users were the least frequent information (Table 4). In open questions, professionals reported that this instrument was instituted by residency programs in two public hospital units in the network and that its use was already well established in most private hospitals, as shown in Table 4.

All PHC physicians, with some frequency, participated in training on protocols. Most of them considered that the institution provided time to participate in clinical sessions, whose content was considered appropriate. The number of specialists that knew such instruments was lower, with the participation of specialists in clinical sessions being almost zero.

### Difficulties in Using Coordination Mechanisms between Healthcare Levels

For physicians from PHC and SC, the mechanism that presented the greatest difficulties of use was the shared protocols, due to the barriers to perform the tests and the unavailability of standardized supplies. More than half of the specialists reported difficulties to participate in clinical sessions, with lack of time being pointed out as the main difficult (Table 5).

**Table 5:** Difficulties in Using Coordination Mechanisms between Levels of Service Medium-size municipality, Northeast, Brazil, 2019.

		PHC n (%)	SC n (%)	Total n (%)	p
**Difficulties**					
**Referral form and reply letters**		**n = 55**	**n = 41**	**n = 96**	0.916
	Yes	18 (32.7)	13 (31.7)	31 (32.3)	
	No	37 (67.3)	28 (68.3)	65 (67.7)	
**Hospital discharge report**		**n = 50**	-	-	-
	Yes	13 (26.0)	-	-	
	No	37 (74.0)	-	-	
**Shared protocols**		**n = 42**	**n = 22**	**n = 64**	0.586
	Yes	18 (42.9)	11 (50.0)	29 (45.3)	
	No	24 (57.1)	11 (50.0)	35 (54.7)	
**Clinical sessions**		**n = 29**	**n = 9**	**n = 38**	0.436
	Yes	10 (34.5)	5 (55.6)	15 (39.5)	
	No	19 (65.5)	4 (44.4)	23 (60.5)	

PHC: primary health care; SC: specialized care.

Note: Questions answered only by professionals that knew the existence of the instrument, according to Table 3.

Table 5 shows that most participants found no barriers in using the referral and reply letters and the discharge report. In open questions, most respondents pointed out the fact that these documents are sent via patient as a reason for not receiving them. Lack of time to fill in the forms due to physicians' workload was mentioned by PHC and SC physicians as a difficulty. The interviewees' statements also referred to the low functionality (objectivity) of the printed material and the lack of standardization, management request and the physicians' low interest.

## DISCUSSION

Only mechanisms known to professionals in their daily practice could be used to obtain better coordination. Feedback instruments such as referral and reply letters, hospital discharge report and WhatsApp are widely known by professionals of both levels. Clinical sessions and protocols were little known, especially in SC.

Knowing is a necessary condition; however, it is not enough to include such mechanisms in the work process. The traditional referral forms and reply letters, although available to most of family health teams in the country^18^, had low or no frequency of use, especially in SC, although difficulties in using them have not been identified.

Our study confirms that reply letters was not used as a feedback mechanism, evidence frequently reiterated by a set of studies^4^^,^^14^, considering also the experience of specialists, an actor little incorporated in the studies on coordination. Oliveira et al.^4^ draw attention to the bureaucratic character in understanding this mechanism, despite being the most common and, often, the only one found in the HCN. Nevertheless, few doctors considered it for longitudinal monitoring. In the case of PHC, the delay in the arrival of the reply letters may delay actions of active search in cases that require more immediate interventions. The lower frequency of information about exams in the instrument also reduces the chances of reducing unnecessary duplication^12^, an aspect to be considered by management in training processes.

Insofar as the regulatory centers did not mediate the flow of clinical information, but rather the users themselves, non-institutionalization prevailed, which, in turn, could interfere with the professionals' decision to use it. This result is compatible with those found in other studies, which also identified a prevalent pattern of referral and reply letters sending via users in networks with different characteristics^4^.

Another feedback tool, the hospital discharge report, despite dealing with the relationship with hospital services, was well known, and an important percentage of PHC physicians received it. In the municipality, the experience of implantation via medical residency in public hospitals has made the discharge report present in the PHC work process, reinforcing the premise that coordination mechanisms must be part of every point of the care service^12^. Likewise, such experience, triggered by a training program, seems to have been decisive in making receiving it more frequent than reply letters, even though driving via the user remained, the lack of information and indications for monitoring in PHC and low sharing of test results, a finding also found in another study^4^. The set of results seems to indicate that, in addition to the institutionalization of coordination mechanisms, their use by professionals includes the recognition of networking and PHC as a regular search and longitudinal care service^14^, which can be facilitated by training processes in the undergraduate course and in professional action^9^.

Although Telehealth is a strategy in the scope of information and communication technologies to promote greater access, improve quality and professional training implemented in the country since 2007 to connect PHC to other levels^19^, it was unknown by SC physicians. Electronic referral means (or e-referrals) can decrease the time to access the specialist, reduce costs and improve coordination, with a greater degree of success if implemented in networks with salaried specialists^20^, such as the municipality of study. Among the devices offered by *Telessaúde Brasil Redes*, telediagnosis, in which exams are sent to specialists for issuing reports, could be a better explored strategy.

WhatsApp, a mutual adjustment mechanism for informal communication^11^, was widely known, but used to contact only known professionals, which also signals that close relationships are necessary for collaboration^8^^,^^21^. Countryside areas also presented internet access problems.

In PHC, investments in computerization with the implementation of electronic medical records have been observed in recent years^18^. In the municipality, since 2018, the HealthRise Project, in partnership with public universities, has expanded the implementation of the citizen's electronic medical record in the FHS, which may explain the greater knowledge of the mechanism at this level. However, there was no progress in relation to shared medical records, vertical information system^12^, present in only 14% of primary care teams in the country, especially in municipalities with more than 100,000 inhabitants^22^. Because they are not shared, in the context of small and independent providers, professionals are unable to access clinical records in the various services of the HCN^20^.

Among the coordination mechanisms by standardization^12^, shared protocols and clinical sessions were even more residual, mainly in SC. A study conducted in two HCN in the state of Pernambuco also showed low knowledge about the protocols prepared by the Brazilian Ministry of Health by SC physicians^4^, which seems to question their “sharing” nature. Although less well known than the feedback instruments, there was high use, more expressive in PHC, that is, those that knew used the protocols to standardize the care, as found by Oliveira et al.^4^ At the same time, professionals pointed out difficulties in incorporating the protocol guidelines, since the HCN did not offer the necessary supplies and services. Clinical sessions, on the other hand, were not part of the SC work process, a result also found in another study^4^, and were partially present in the PHC, requiring a guarantee of time, by the management, to enable adherence to this coordination mechanism^9^.

Our study did not analyze possible associations between knowledge and the use of coordination mechanisms by physicians, nor did it cover other actors and sources of information that might need its implementation and use in the HCN, such as monitoring references, frequency of holding clinical sessions and making available protocols, among others. Our study used an instrument applied in national and international scenarios, which proved to be adequate for the identification and in-depth analysis of coordination mechanisms, characterizing and qualifying aspects of use that can be appropriated by researchers and health managers involved in the improvement coordination of care service.

## CONCLUSIONS

PHC attributions has been increasing, with the successive incorporation of care services previously provided at other levels^20^ and, in the Brazil, decentralization of actions and programs^23^, in addition to increased care overload, mainly attributed to insufficient human resources^9^ and an excess of users per team^14^. Problems related to the insufficient supply of services in the HCN^14^ also discourage and hinder the use of standardization mechanisms. In this scenario, there seems to be a consensus that coordination activities will not arise spontaneously^20^ without a guarantee of adequate working conditions such as sufficient time, payment for performance of coordination activities^20^ and stable employment bonds that allow professionals to develop them, as well as training processes aimed at networking and recognizing PHC role^8^^,^^9^.

Network formation and connection between workers is imperative in health care, since there is no self-sufficiency^24^. The involvement of all SUS workers, including in the investigative processes, seems to be the most promising and possible way, since mutual adjustment mechanisms facilitate the approximation and direct communication among professionals as well as contributing to increase interprofessional trust^12^. In some cases, such mechanisms are characterized as lightweight technologies, which could be encouraged without the need for large resources.

Finally, coordination mechanisms have great potential to improve interprofessional collaboration^2^. Furthermore, as important as implementing those that do not yet exist (such as shared electronic medical records) is to create adequate conditions for the use of such mechanisms, many of which are already present in the HCN, as showed in our study. It is in the complementarity of measures that make it possible to know, develop professional skills, institutionalize and promote organizational conditions for the effective use of coordination mechanisms in all HCN that the fragmentation of the system and care service can be faced.

## Footnotes

a Samico I, Albuquerque PC, Vázquez ML, Vargas I, Unger JP, De Paepe P, et al. Questionário de articulação/coordenação entre os níveis de atenção: COORDENAR-BR. Recife, PE: Instituto de Medicina Integral Prof. Fernando Figueira (IMIP); 2015 [cited 2018 Jul 9]. Available from: http://www.equity-la.eu/upload/seccions/files/COORDENA_BR%282%29.pdf
