# Supplementary material for: Do primary and specialized care physicians know and use coordination mechanisms?
Source: Rev Saude Publica. 2020 Nov 17;54:121. doi: 10.11606/s1518-8787.2020054002475 (PMC7671583; doi:10.11606/s1518-8787.2020054002475)
Supplement: Translation

# Translation: Médicos da atenção primária e especializada conhecem e utilizam mecanismos de coordenação?

**Authors:** Lívia dos Santos Mendes, Patty Fidelis de Almeida

**Affiliations:** I Vitória da ConquistaBA Brasil Universidade Federal da Bahia. Instituto Multidisciplinar em Saúde. Pós-Graduação em Saúde Coletiva. Vitória da Conquista, BA, Brasil; II NiteróiRJ Brasil Universidade Federal Fluminense. Instituto de Saúde Coletiva. Niterói, RJ, Brasil

**Keywords:** Colaboração Intersetorial, Assistência Integral à Saúde, Níveis de Atenção à Saúde, Administração de Serviços de Saúde

## Abstract

**OBJETIVOS:**

Analisar se médicos da atenção primária à saúde e da atenção especializada conhecem e utilizam mecanismos de coordenação do cuidado entre níveis assistenciais.

**MÉTODOS:**

Estudo transversal, do tipo inquérito, com aplicação do instrumento COORDENA-BR a médicos da atenção primária e da atenção especializada da rede pública de um município de médio porte, no período de junho a outubro de 2019. Foi abordado o conhecimento, frequência de envio e recebimento, finalidade, características e dificuldades para a utilização dos mecanismos de *feedback* ou adaptação mútua e de padronização para promoção de coordenação do cuidado entre níveis assistenciais.

**RESULTADOS:**

Instrumentos de *feedback* como formulários de referência e contrarreferência, resumo de alta hospitalar e *WhatsApp* são amplamente conhecidos por profissionais dos dois níveis, sem diferenças significativas. Sessões clínicas e protocolos são pouco reconhecidos, sobretudo na atenção especializada, o que pressupõe baixa utilização de mecanismos de padronização para obtenção de maior coordenação do cuidado entre níveis assistenciais. Apesar do elevado reconhecimento e facilidade de uso, instrumentos de *feedback* tradicionais como guias de referência e contrarreferência não são amplamente utilizados. Menor frequência de médicos conhecia os protocolos, principalmente na atenção especializada, e destacaram dificuldades para sua aplicação, como insuficiência de exames e indisponibilidade de insumos na rede. As sessões clínicas eram pouco conhecidas e tinham baixa frequência de participação. Pressão assistencial, baixa institucionalização e falta de tempo foram barreiras identificadas para a incorporação dos mecanismos de coordenação do cuidado ao processo de trabalho na atenção primária e especializada, além daquelas relacionadas à oferta de serviços na rede.

**CONCLUSÃO:**

Argumenta-se que a fragmentação do sistema e dos cuidados poderá ser enfrentada na complementariedade de medidas que possibilitem conhecer, desenvolver habilidades profissionais, institucionalizar e promover condições organizacionais para a efetiva utilização de mecanismos de coordenação em toda rede de atenção à saúde.

## INTRODUÇÃO

A diversidade e a complexidade das necessidades de saúde diante da transição nutricional, epidemiológica e demográfica aliadas aos avanços tecnológicos incorporados à assistência promoveram, de certa forma, ampliação do arsenal terapêutico disponível e configuraram um padrão de múltiplos contatos com profissionais e serviços de saúde, sobretudo nos casos de multimorbidade^1^^,^^2^, ampliando a necessidade de estratégias para a coordenação do cuidado. O uso de instrumentos para a comunicação e articulação do cuidado entre níveis assistenciais fortalece a coerência, eficiência e qualidade da atenção^3^. A despeito do consenso em torno de sua necessidade, um conjunto de evidências sinaliza incipiência no conhecimento e utilização de mecanismos de coordenação por profissionais em diferentes contextos de sistemas de saúde^4^^,^^5^.

A duplicação de procedimentos diagnósticos e terapêuticos, encaminhamentos desnecessários e ausência de conciliação de tratamentos são ao mesmo tempo resultado e uma das facetas que revelam a ausência de coerência entre níveis assistenciais e a baixa qualidade do cuidado^6^. A falta de interação e de confiança entre os profissionais da atenção primária à saúde (APS) e da atenção especializada (AE) são parte do problema^7^^,^^8^, cujo enfrentamento não pode depender apenas da intenção e iniciativa dos profissionais de saúde, sendo dependente das condições laborais, organizacionais, relacionais e das características do sistema como um todo^9^.

Os instrumentos de coordenação têm por objetivo minimizar as barreiras entre os níveis assistenciais favorecendo a continuidade por meio da transferência de informações, troca de conhecimentos e aperfeiçoamento do cuidado^10^^,^^11^. Mecanismos de coordenação podem ser classificados como de padronização e de *feedback* ou adaptação mútua^11^^,^^12^. Os primeiros objetivam a harmonização ou sistematização do processo de trabalho, das habilidades dos profissionais e dos resultados do trabalho, como sessões clínicas e protocolos compartilhados. Os de adaptação mútua ou *feedback* são baseados na comunicação e na troca de informação formal e informal entre profissionais para o planejamento compartilhado do cuidado, como formulários de referência e contrarreferência, resumo de alta hospitalar e telefone^12^. Esses instrumentos são importantes para a promoção de ajustes em situações clínicas que envolvem elevado conjunto de informações, atividades altamente especializadas e, ao mesmo tempo, interdependentes^13^.

O emprego adequado de mecanismos de coordenação seria um dos facilitadores da comunicação e integração entre APS e AE^4^^,^^14^. Estudos nacionais sobre o tema frequentemente têm nas equipes e nos médicos da APS o foco das análises^14^^,^^15^, uma vez que, nas definições e diretrizes das políticas nacionais, cabe a esse nível de atenção a coordenação do cuidado nas redes de atenção à saúde (RAS). A partir da experiência e percepção dos dois principais atores envolvidos nas atividades de articulação da atenção entre os distintos serviços da RAS, este artigo objetiva analisar se médicos da APS e da AE conhecem e utilizam mecanismos de coordenação do cuidado entre níveis assistenciais.

## MÉTODOS

Trata-se de um estudo transversal, do tipo inquérito, realizado em serviços de APS e AE da rede do Sistema Único de Saúde (SUS) de um município de médio porte (338.480 habitantes em 2019) do estado da Bahia, Brasil. Foi realizado censo dos profissionais médicos da APS e da AE em atuação nos serviços, entre os meses de junho e outubro de 2019. A APS apresentava cobertura de 60%, sendo 47% pela Estratégia Saúde da Família (ESF) e 13% por unidades básicas de saúde (UBS) tradicionais^16^.

Fizeram parte da amostra todos os médicos da APS (ESF e UBS tradicionais) e especialistas de serviços que recebiam referências regulares da atenção primária para consultas especializadas. Os participantes trabalhavam há pelo menos três meses no respectivo serviço no período de início do campo, segundo informações da gestão municipal. Foram entrevistados 120 dos 136 médicos. Sujeitos entrevistados e perdas, conforme serviço de atuação, estão descritos na Tabela 1.

**Tabela 1:** Médicos da atenção primária à saúde e da atenção especializada entrevistados de acordo com serviço de saúde de atuação. Município de médio porte, Nordeste, Brasil, 2019.

Tipo de serviço			Número de médicos	Número de perdas	Número de entrevistados
APS	ESF	Zona rural	16	2	14
		Zona urbana	28	2	26
	**Total de médicos da ESF**		**44**	**4**	**40**
	**Total de Médicos de UBS**		**26**	**2**	**24**
**Total de médicos da APS**			**70**	**6**	**64**
AE	Centro de especialidades médicas		52	9	43
	Ambulatório de saúde mental		5	-	5
	Clínica de reabilitação		2	-	2
	UBS		7	1	6
**Total de médicos especialistas**			**66**	**10**	**56**
**Total**			**136**	**16**	**120**

APS: atenção primária à saúde; AE: atenção especializada; ESF: Estratégia Saúde da Família; UBS: unidades básicas de saúde.

Para realização do estudo, foi utilizado o instrumento validado e traduzido para o português COORDENA-BR^a^, que se baseia no modelo teórico desenvolvido por Vázquez et al.^2^ e Vargas et al.^9^ para análise da coordenação do cuidado entre níveis assistenciais. O questionário completo aborda: experiência de coordenação da informação e gestão da clínica entre níveis assistenciais e percepção geral sobre a coordenação entre níveis na rede; fatores de interação dos profissionais; conhecimento e uso de mecanismos de coordenação; sugestões para a melhoria; fatores organizacionais, laborais e atitudes relacionados à coordenação entre níveis; dados sociodemográficos dos entrevistados. Os itens referentes ao conhecimento e uso de mecanismos de coordenação entre níveis são o foco deste artigo.

No questionário são abordados o conhecimento, frequência de envio e recebimento, finalidade, características e dificuldades para a utilização dos mecanismos de *feedback* (ou adaptação mútua) e de padronização para promoção de coordenação do cuidado entre níveis assistenciais, que compuseram as variáveis analisadas. O questionário sofreu pequenas adequações ao cenário local (por exemplo, o tipo de protocolo adotado na RAS) e algumas atualizações, como o acréscimo de uma questão sobre conhecimento acerca do prontuário eletrônico e Telessaúde implantados no município, segundo informações da gestão, e *WhatsApp*, tecnologia cada vez mais utilizada na comunicação interprofissional^17^. A finalidade do uso e as dificuldades encontradas foram identificadas por meio de perguntas abertas. Do mesmo modo, havia campos abertos para explicar os motivos do não recebimento regular dos formulários de referência, contrarreferência e do resumo de alta hospitalar.

Para as repostas fechadas, foram utilizadas a escala de Likert e respostas dicotômicas (sim/não), além de questões de múltipla escolha. Algumas variáveis de interesse foram categorizadas para melhor comparação entre médicos da APS e da AE. As respostas “sempre” e “muitas vezes” bem como “diariamente” e “semanalmente” foram consideradas como alta frequência; “às vezes” e “muito poucas vezes” assim como “mensalmente” e “com menor frequência” como baixa frequência; e “nunca” representou a não utilização do instrumento (frequência nula). O questionário foi digitalizado utilizando-se o *software KoBo Toolbox 1.4.8*, disponível em *tablets,* marca Samsung, modelo Galaxy Tab A.

A coleta de dados foi realizada por meio de entrevistas presenciais realizadas por pesquisadores capacitados, com duração média de 26,6 minutos, sendo gravadas em aparelho de áudio para transcrição e categorização das questões abertas. Para garantia da qualidade da coleta e confiabilidade dos dados, procedeu-se ao monitoramento direto das atividades de campo e a avaliação do preenchimento de todos os questionários no banco de dados.

Foi realizada análise descritiva das variáveis, por nível assistencial, por meio de frequências absolutas (n) e relativas (%). Os dados foram processados no programa *Stata*, versão 15.0 (*Stata Corporation, College Station, USA*). As diferenças entre as proporções foram avaliadas pelo teste do qui-quadrado de Pearson e pelo teste exato de Fisher. Os dados qualitativos, procedente das respostas abertas, foram categorizados.

O estudo foi aprovado pelo Comitê de Ética em Pesquisa da Universidade Federal da Bahia, sob parecer n° 3.334.464 e CAAE: 09503419.1.0000.5556 com anuência do município.

## RESULTADOS

### Características da Amostra

A maior proporção dos profissionais era do sexo masculino (57%), sendo na APS a maioria mulheres (55%), mais jovens, na faixa etária de 25 a 34 anos (40%). Os médicos da AE eram formados há mais tempo e a maioria (55%) era de egressos de universidades públicas. Na APS predominou a formação em faculdades privadas e cerca de 60% não tinham formação em residência médica. A grande maioria dos especialistas possuía vínculos trabalhistas com menor carga horária semanal (80%) e atuava também no setor privado (93%), diferentemente da APS (Tabela 2).

**Tabela 2:** Caracterização da amostra. Município de médio porte, Nordeste, Brasil, 2019.

Características		APS (n = 64) n (%)	AE (n = 56) n (%)	Total (n = 120) n (%)
Sexo				
	Feminino	35 (54,7)	17 (30,4)	52 (43,3)
	Masculino	29 (45,3)	39 (69,6)	68 (56,7)
Idade (anos)				
	25–34	26 (40,6)	11 (19,6)	37 (30,8)
	35–49	22 (34,4)	34 (60,7)	56 (46,7)
	> 50	16 (25,0)	11 (19,6)	27 (22,5)
Nacionalidade				
	Brasil	64 (100,0)	56 (100,0)	120 (100,0)
Tempo de formação (anos)				
	≤ 2	15 (23,4)	0 (0,0)	15 (12,5)
	3–10	26 (40,6)	15 (26,8)	41 (34,2)
	11–20	7 (10,9)	24 (42,8)	31 (25,8)
	> 20	16 (25,0)	17 (30,4)	33 (27,5)
Instituição de formação				
	Pública	24 (37,5)	31 (55,4)	55 (45,8)
	Particular	40 (62,5)	25 (44,6)	65 (54,2)
Especialização médica				
	Sem residência médica	38 (59,4)	1 (1,8)	39 (32,5)
	Clínica médica	9 (14,1)	0 (0,0)	9 (7,0)
	Pediatria	7 (10,9)	5 (8,9)	12 (10,0)
	Ginecologia/obstetrícia	3 (4,7)	6 (10,7)	9 (7,0)
	Outros	7 (10,9)	44 (78,6)	51 (42,5)
Horas de trabalho por semana				
	20–32	42 (65,6)	45 (80,4)	87 (72,5)
	≥ 40	22 (34,4)	11 (19,6)	33 (27,5)
Trabalha no setor privado				
	Sim	31 (48,4)	52 (92,9)	83 (69,2)
	Não	33 (51,6)	4 (7,1)	37 (30,8)

APS: atenção primária à saúde; AE: atenção especializada.

### Conhecimento e Utilização dos Mecanismos de Coordenação do Cuidado entre Níveis Assistenciais

O mecanismo formal de coordenação entre níveis mais conhecido entre os médicos da APS foi o formulário de referência e contrarreferência (86%), seguido do resumo de alta hospitalar (78%). Os especialistas conheciam mais o resumo de alta (91%), seguido do formulário de referência e contrarreferência (70%). Com relação aos instrumentos de padronização, os protocolos compartilhados entre níveis, elaborados pelo Ministério da Saúde e pelo município, eram mais conhecidos pelos médicos da APS quando comparados aos especialistas (p = 0,004). As sessões clínicas conjuntas entre médicos da APS e da AE eram pouco conhecidas por ambos, sendo que os especialistas praticamente as desconheciam (p = 0,001), conforme apresentado na Tabela 3.

**Tabela 3:** Existência e frequência de utilização dos mecanismos de coordenação entre níveis assistenciais. Município de médio porte, Nordeste, Brasil, 2019.

		APS (n = 64) n (%)	AE (n = 56) n (%)	Total (n = 120) n (%)	p
Existência do instrumento					
Formulário de referência e contrarreferência					
	Sim	55 (85,9)	41 (73,2)	96 (80,0)	0,082
	Não	9 (14,1)	15 (26,8)	24 (20,0)	
Resumo de alta hospitalar					
	Sim	50 (78,1)	51 (91,1)	101 (84,2)	0,053
	Não	14 (21,9)	5 (8,9)	19 (15,8)	
Protocolos do MS e municipais compartilhados entre níveis					
	Sim	42 (65,6)	22 (39,3)	64 (53,3)	**0,004**
	Não	22 (34,4)	34 (60,7)	56 (46,7)	
Sessões clínicas conjuntas entre médicos da APS e da AE					
	Sim	29 (45,3)	9 (16,1)	38 (31,7)	**0,001**
	Não	35 (54,7)	47 (83,9)	82 (68,3)	
Telefone institucional					
	Sim	41 (64,1)	51 (91,1)	92 (76,7)	**< 0,001**
	Não	23 (35,9)	5 (8,9)	28 (23,3)	
Telessaúde					
	Sim	35 (54,7)	5 (8,9)	40 (33,3)	**< 0,001**
	Não	29 (45,3)	51 (91,1)	80 (66,7)	
Prontuário eletrônico					
	Sim	29 (45,3)	6 (10,7)	35 (29,2)	**< 0,001**
	Não	35 (54,7)	50 (89,3)	85 (70,8)	
*WhatsApp*					
	Sim	56 (87,5)	54 (96,4)	110 (91,7)	0,102
	Não	8 (12,5)	2 (3,6)	10 (8,3)	
Frequência^a^					
Envio do formulário de referência e contrarreferência^b^		n = 55	n = 41	n = 96	**0,038**
	Alta	24 (43,6)	8 (19,5)	32 (33,3)	
	Baixa	22 (40,0)	21 (51,2)	43 (44,8)	
	Nunca	9 (16,4)	12 (29,3)	21 (21,9)	
Recebimento do formulário de referência e contrarreferência^c^		n = 55	n = 41	n = 96	**0,012**
	Alta	2 (3,6)	7 (17,1)	9 (9,4)	
	Baixa	26 (47,3)	24 (58,5)	50 (52,1)	
	Nunca	27 (49,1)	10 (24,4)	37 (38,5)	
Recebimento do resumo de alta hospitalar		n = 50	n = 51	n = 101	-
	Alta	22 (44,0)	-	-	
	Baixa	25 (50,0)	-	-	
	Nunca	3 (6,0)	-	-	
Utilização de protocolos do MS e municipais compartilhados entre níveis		n = 42	n = 22	n = 64	**0,006**
	Alta	39 (92,8)	14 (63,6)	53 (82,9)	
	Baixa	2 (4,8)	2 (9,1)	4 (6,2)	
	Nunca	1 (2,4)	6 (27,3)	7 (10,9)	
Participação nas sessões clínicas conjuntas entre médicos da APS e da AE		n = 29	n = 9	n = 38	**0,002**
	Alta	19 (65,5)	1 (11,1)	20 (52,6)	
	Baixa	10 (34,5)	6 (66,7)	16 (42,1)	
	Nunca	0 (0,0)	2 (22,2)	2 (5,3)	
*Uso do WhatsApp*		n = 56	n = 54	n = 110	0,259
	Alta	7 (12,5)	2 (3,7)	9 (8,2)	
	Baixa	22 (39,3)	22(40,7)	44 (40,0)	
	Nunca	27 (48,2)	30 (55,6)	57 (51,8)	

APS: atenção primária à saúde; AE: atenção especializada; MS: Ministério da Saúde.

a Questões respondidas apenas pelos profissionais que reconheceram a existência do instrumento.

b O envio do formulário de referência foi respondido pelos médicos da APS e o da contrarreferência pelos médicos da AE.

c O recebimento do formulário de referência foi respondido pelos médicos da AE e o da contrarreferência pelos médicos da APS.

Valores com significância estatística estão apresentados em negrito.

Cerca de 36% dos médicos da APS não reconhecia a existência de telefone institucional e 45% desconheciam o Telessaúde. Entre os médicos AE, o telefone era amplamente conhecido e o Telessaúde desconhecido. O prontuário eletrônico parecia estar presente em parte dos serviços de APS, mas ausente na AE, já que a grande maioria não o conhecia. O *WhatsApp* foi o instrumento mais conhecido pelos profissionais dos dois níveis. Houve diferença significativa entre o conhecimento dos médicos da APS e da AE acerca da maioria dos mecanismos de coordenação do cuidado (Tabela 3).

Após o reconhecimento da existência, buscou-se compreender a frequência da utilização das ferramentas de coordenação, categorizada como alta, baixa ou nunca. A maioria dos profissionais reconheceu os instrumentos de referência e contrarreferência. Contudo, apenas 44% dos médicos da APS mencionaram alta frequência no envio da referência ao especialista, que, por sua vez, mencionou baixa frequência no recebimento. Por outro lado, menor número de especialistas reconhecia tais instrumentos e poucos desses enviavam a contrarreferência frequentemente aos médicos da APS, dos quais cerca da metade relatou nunca a receber. O recebimento do resumo de alta hospitalar pelos médicos do primeiro nível era mais frequente. O fato de estes instrumentos serem transportados pelos pacientes foi bastante mencionado pelos entrevistados como motivo para o não recebimento. Entre os médicos da APS que conheciam os protocolos compartilhados (42), a grande maioria apresentava alta frequência de uso desta ferramenta (93%). Entre os especialistas, a minoria a reconhecia (22); desses, 64% a utilizavam com alta frequência. As sessões clínicas eram pouco conhecidas por ambos e ainda menos utilizadas por especialistas. O *WhatsApp* era amplamente reconhecido pelos profissionais (mais frequente nos serviços de AE), mas com baixa ou nenhuma utilização (Tabela 3).

### Finalidade e Características da Utilização dos Mecanismos de Coordenação entre Níveis Assistenciais

Entre os profissionais que reconheciam os mecanismos e mencionaram utilizá-los com alguma frequência, foi avaliada sua finalidade por meio de perguntas abertas cujo conteúdo foi categorizado. A grande maioria considerou que os formulários de referência, contrarreferência e resumo de alta hospitalar eram utilizados para a troca de informação entre níveis. Para os médicos da APS e da AE, os protocolos tinham como principal função orientar o atendimento e padronizar a atenção, sendo referida ainda a qualidade da atenção e a redução de custos. Na opinião dos poucos médicos que reconheciam as sessões clínicas, os principais objetivos seriam aprimorar o conhecimento e unificar condutas (Tabela 4).

**Tabela 4:** Finalidade e características da utilização dos mecanismos de coordenação entre níveis assistenciais. Município de médio porte, Nordeste, Brasil, 2019.

			APS n (%)	AE n (%)	Total n (%)
Finalidade^a^ ^b^					
Formulário de referência e contrarreferência			n = 46	n = 29	n = 75
	Trocar informação		38 (82,6)	28 (96,5)	66 (88,0)
	Encaminhar o paciente		10 (21,7)	1 (3,4)	11 (14,7)
	Acompanhamento na APS		2 (4,3)	9 (31,0)	11 (14,7)
	Agilizar o acesso ao especialista		4 (8,7)	0 (0,0)	4 (5,3)
	Burocracia		2 (4,3)	1 (3,4)	3 (4,0)
Resumo de alta hospitalar			n = 47	-	-
	Trocar informação		35 (74,5)	-	-
	Acompanhamento na APS		17 (36,2)	-	-
Protocolos compartilhados			n = 41	n = 16	n = 57
	Orientar o atendimento		19 (46,3)	9 (56,2)	28 (49,1)
	Padronizar a atenção		19 (46,3)	7 (43,7)	26 (45,6)
	Qualidade da atenção		6 (14,6)	1 (6,2)	7 (12,3)
	Reduzir custos		1 (2,4)	2 (12,5)	3 (5,3)
	Favorecer a articulação		3 (7,3)	0 (0,0)	3 (5,3)
	Evitar encaminhamentos desnecessários		3 (7,3)	0 (0,0)	3 (5,3)
	Burocracia		1 (2,4)	0 (0,0)	1 (1,7)
Sessões clínicas			n = 29	n = 7	n = 36
	Aprimorar conhecimento		17 (58,6)	3 (42,8)	20 (55,5)
	Unificar conduta		13 (44,8)	4 (57,1)	17 (47,2)
	Discussão de casos clínicos		5 (17,2)	4 (57,1)	9 (25,0)
	Troca de experiências		3 (10,3)	3 (42,8)	6 (16,7)
*WhatsApp*			n = 29	n = 24	n = 53
	Facilidade/rapidez		17 (58,6)	21 (87,5)	38 (71,7)
	Trocar informações		11 (37,9)	13 (54,2)	24 (45,3)
	Agilizar o acesso ao especialista		6 (20,7)	1 (4,2)	7 (13,2)
Características da utilização					
Formulário de referência e contrarreferência^a^					
	Como envia os formulários de referência e contrarreferência		n = 46	n = 29	n = 75
		Pelo paciente	37 (80,4)	28 (96,6)	65 (86,7)
		Pela central de regulação	9 (19,6)	1 (3,4)	10 (13,3)
	Como recebe os formulários de referência e contrarreferência		n = 28	n = 31	n = 59
		Pelo paciente	27 (96,4)	29 (93,5)	56 (94,9)
		Pela central de regulação	1 (3,6)	2 (6,5)	3 (5,1)
	Informações que recebe na referência^c^		-	n = 31	-
		Tratamentos	-	20 (64,5)	-
		Diagnóstico	-	20 (64,5)	-
		Dados pessoais	-	29 (93,5)	-
		História clínica	-	24 (77,4)	-
		Exames	-	18 (58,0)	-
		Motivo do encaminhamento	-	31 (100,0)	-
	Informações que recebe na contrarreferência^c^		n = 28	-	-
		Tratamentos	25 (89,3)	-	-
		Diagnóstico	26 (92,9)	-	-
		Dados pessoais	19 (67,9)	-	-
		História clínica	18 (64,3)	-	-
		Exames	17 (60,7)	-	-
	A contrarreferência responde ao motivo da referência.		n = 28	-	-
		Alta	16 (57,1)	-	-
		Baixa	10 (35,7)	-	-
		Nunca	2 (7,2)	-	-
	A contrarreferência é recebida em tempo oportuno para tomar decisões.		n = 28	-	-
		Alta	9 (32,1)	-	-
		Baixa	15 (53,6)	-	-
		Nunca	4 (14,3)	-	-
Resumo de alta hospitalar^a^					
	Recebe a informação em tempo oportuno para tomar decisões.		n = 47	-	-
		Alta	16 (34,0)	-	-
		Baixa	29 (61,7)	-	-
		Nunca	2 (4,3)	-	-
	Como recebe o resumo de alta hospitalar		n = 47	-	-
		Pelo paciente	47 (100,0)	-	-
	Informações que recebe no resumo de alta hospitalar^c^		n = 47	-	-
		Tratamento farmacológico	47 (100,0)	-	-
		Diagnóstico	47 (100,0)	-	-
		Motivo de internação	46 (97,9)	-	-
		Procedimentos realizados	47 (100,0)	-	-
		Resultados de exames	40 (85,1)	-	-
		Indicações para o acompanhamento	30 (63,8)	-	-
Protocolos compartilhados^d^					
Participa de capacitações para utilização dos protocolos compartilhados			n = 42	n = 22	n = 64
	Alta		27 (64,3)	8 (36,4)	35 (54,7)
	Baixa		15 (35,7)	14 (63,6)	29 (45,3)
	Nunca		0 (0,0)	0 (0,0)	0 (0,0)
Sessões clínicas^d^					
A instituição proporciona tempo para participar das sessões clínicas			n = 29	n = 9	n = 38
	Sim		20 (69,0)	4 (44,5)	24 (63,2)
	Às vezes		6 (20,7)	2 (22,2)	8 (21,0)
	Não		3 (10,3)	1 (11,1)	4 (10,5)
	Não são realizadas com a minha especialidade		0 (0,0)	2 (22,2)	2 (5,3)
	Conteúdo		n = 29	n = 7	n = 36
		Adequado	27 (93,1)	7 (100,0)	34 (94,4)

APS: atenção primária à saúde; AE: atenção especializada.

a n definido pelo número de profissionais que apresentaram alguma frequência de utilização ou recebimento do instrumento, conforme a Tabela 3.

b Questões abertas e categorizadas.

c Questões de múltipla escolha que admitiam mais de uma resposta.

d n definido pelo número de profissionais que reconheceram a existência do instrumento, conforme a Tabela 3.

O uso do telefone e *WhatsApp* foi justificado pela facilidade e rapidez, embora a maioria não os utilizasse (Tabela 3). Muitos médicos da APS relataram que esse instrumento de comunicação poderia agilizar o acesso ao especialista nas situações de maior urgência, além de garantir que a informação chegasse ao outro nível (Tabela 4), o que não ocorria no caso da referência e contrarreferência conduzidas pelo usuário. Os dados qualitativos revelaram que os médicos usavam telefone e *WhatsApp* para entrar em contato apenas com profissionais que já conheciam anteriormente. Médicos da APS que trabalhavam em unidades de zona rural ressaltaram que o acesso à internet era viabilizado pelo próprio profissional.

Em relação às características da utilização dos mecanismos de coordenação, na referência preenchida pelos médicos da APS, os especialistas recebiam mais informações sobre motivos do encaminhamento (100%), dados pessoais e história clínica. Na contrarreferência realizada por especialistas, médicos do primeiro nível recebiam mais informações sobre tratamentos e diagnóstico (Tabela 4). Resultados de exames foram as informações menos frequentes. Entre os médicos da APS que recebiam a contrarreferência (28), metade considerava que respondia ao motivo do encaminhamento e só um terço que chegava em tempo oportuno. Ainda assim, mais da metade dos especialistas não conheciam ou não utilizavam tais instrumentos.

Segundo a maioria dos médicos da APS, os resumos de alta hospitalar também não eram recebidos em tempo oportuno para a tomada de decisões. A totalidade considerou que neste instrumento estavam presentes informações sobre tratamento farmacológico, diagnóstico e procedimentos realizados. Contudo, as indicações para o acompanhamento dos usuários foi a informação menos frequente (Tabela 4). Em perguntas abertas, profissionais informaram que esse instrumento foi instituído pelos programas de residência em duas unidades hospitalares públicas da rede e que seu uso já estava bem consolidado na maioria dos hospitais privados, como pode ser observado na Tabela 4.

Todos os médicos da APS, com alguma frequência, participaram de capacitações sobre protocolos. A maioria considerou que a instituição proporcionou tempo para participar de sessões clínicas, cujo conteúdo foi considerado adequado. O número de especialistas que conheciam tais instrumentos era menor, sendo quase nula a participação dos especialistas nas sessões clínicas.

### Dificuldades na Utilização dos Mecanismos de Coordenação entre Níveis Assistenciais

Para médicos da APS e da AE, o mecanismo que apresentava maiores dificuldades para utilização eram os protocolos compartilhados, em função das barreiras para a realização dos exames e indisponibilidade de insumos padronizados. Pouco mais da metade dos especialistas referiu dificuldades para participar das sessões clínicas, sendo a falta de tempo a principal (Tabela 5).

**Tabela 5:** Dificuldade na utilização dos mecanismos de coordenação entre níveis assistenciais. Município de médio porte, Nordeste, Brasil, 2019.

		APS n (%)	AE n (%)	Total n (%)	p-valor
Dificuldades					
Formulário de referência e contrarreferência		n = 55	n = 41	n = 96	
	Sim	18 (32,7)	13 (31,7)	31 (32,3)	0,916
	Não	37 (67,3)	28 (68,3)	65 (67,7)	
Resumo de alta hospitalar		n = 50	-	-	
	Sim	13 (26,0)	-	-	-
	Não	37 (74,0)	-	-	
Protocolos compartilhados entre níveis		n = 42	n = 22	n = 64	
	Sim	18 (42,9)	11 (50,0)	29 (45,3)	0,586
	Não	24 (57,1)	11 (50,0)	35 (54,7)	
Sessões clínicas		n = 29	n = 9	n = 38	
	Sim	10 (34,5)	5 (55,6)	15 (39,5)	0,436
	Não	19 (65,5)	4 (44,4)	23 (60,5)	

APS: atenção primária à saúde; AE: atenção especializada.

Nota: Questões respondidas apenas pelos profissionais que reconheceram a existência do instrumento, segundo a Tabela 3.

A maioria dos participantes não encontrou barreiras na utilização do formulário de referência e contrarreferência e do resumo de alta hospitalar, conforme apresentado na Tabela 5. Grande parte dos respondentes destacou, nas questões abertas, o fato de esses documentos serem conduzidos pelo paciente como motivo para o não recebimento. A falta de tempo para o preenchimento pela sobrecarga de trabalho de médicos da APS e da AE foi mencionada como dificuldade. As falas dos entrevistados aludiram também à baixa funcionalidade (objetividade) do impresso e falta de padronização, de cobrança pela gestão e de interesse dos médicos.

## DISCUSSÃO

Apenas mecanismos conhecidos pelos profissionais em sua prática cotidiana poderiam ser utilizados para obtenção de melhor coordenação. Os resultados mostraram que instrumentos de *feedback* como formulário de referência e contrarreferência, resumo de alta hospitalar, telefone e *WhatsApp* eram amplamente conhecidos por profissionais dos dois níveis. Sessões clínicas e protocolos foram pouco reconhecidos, sobretudo na AE.

Conhecer é condição necessária, porém não suficiente para inserção de tais mecanismos ao processo de trabalho. As tradicionais guias de referência e contrarreferência, embora disponíveis à grande maioria das equipes de saúde da família no país^18^, tiveram baixa ou nenhuma frequência de uso, principalmente na AE, ainda que não tenham sido identificadas dificuldades para utilizá-las.

Este estudo ratifica, considerando também a experiência de especialistas, ator pouco incorporado aos estudos sobre coordenação, que a contrarreferência não era utilizada como mecanismo de *feedback*, evidência frequentemente reiterada por um conjunto de estudos^4^^,^^14^. Oliveira et al.^4^ alertam para o caráter burocrático na compreensão desse mecanismo, apesar de ser o mais comum e, muitas vezes, o único encontrado nas RAS. Ainda assim, poucos médicos o consideraram para o acompanhamento longitudinal. No caso da APS, a demora na chegada da contrarreferência pode protelar ações de busca ativa em casos que demandam intervenções mais imediatas. A menor frequência de informações sobre exames no instrumento também reduz as possibilidades de diminuição da duplicação desnecessária^12^, um aspecto a ser considerado pela gestão em processos formativos.

Na medida em que as centrais de regulação não mediavam o trânsito das informações clínicas, e sim os próprios usuários, prevalecia a não institucionalização, que por sua vez poderia interferir na decisão de utilização por parte dos profissionais. Esse resultado é compatível com os encontrados em outros estudos, que também identificaram padrão prevalente de transporte da referência e contrarreferência via usuários em redes com características distintas^4^.

Outro instrumento de *feedback*, o resumo de alta hospitalar, embora trate da relação com serviços hospitalares, era bastante conhecido, e percentual importante de médicos da APS o recebiam. No município, a experiência de implantação via residência médica em hospitais da rede pública fez com que o resumo de alta estivesse presente no processo de trabalho da APS, reforçando a premissa de que mecanismos de coordenação devem fazer parte de todo ponto de atenção^12^. Nesse mesmo sentido, tal experiência, disparada por aparelho formador, parece ter sido determinante para tornar seu recebimento mais frequente do que a contrarreferência, ainda que permanecessem a condução via usuário, a falta de informações e indicações para o seu acompanhamento na APS e o baixo compartilhamento de resultados de exames, achado também encontrado em outro estudo^4^. O conjunto dos resultados parece indicar que, além da institucionalização de mecanismos de coordenação, seu uso pelos profissionais passa pelo reconhecimento do trabalho em rede e da APS como serviço de busca regular e de cuidado longitudinal^14^, que pode ser facilitado por processos formativos na graduação e em serviço^9^.

Embora o Telessaúde seja uma estratégia no âmbito das tecnologias de informação e comunicação para promoção de maior acesso, melhoria da qualidade e formação profissional implementada no país desde 2007 para conectar a APS aos demais níveis^19^, médicos da AE o desconheciam. Esquemas de referência eletrônica (ou e-referência) têm potencial para diminuir o tempo de acesso ao especialista, reduzir custos e melhorar a coordenação, com maior grau de sucesso se implementados em redes nas quais os especialistas sejam assalariados^20^, como o município de estudo. Entre os dispositivos oferecidos pelo Telessaúde Brasil Redes, o telediagnóstico com envio de exames a especialistas para emissão de laudos poderia ser uma estratégia mais bem explorada.

O *WhatsApp*, mecanismo de ajustamento mútuo por comunicação informal^11^, era amplamente conhecido, mas utilizado para entrar em contato apenas com profissionais conhecidos, o que mais uma vez sinaliza que relações de proximidade são necessárias à colaboração^8^^,^^21^. Na zona rural, eram acrescidos problemas de acesso à internet.

Na APS observa-se, nos últimos anos, investimentos em informatização com implementação de prontuários eletrônicos^18^. No município, desde 2018, o Projeto *HealthRise*, em parceria com universidades públicas, ampliou a implantação do prontuário eletrônico do cidadão na ESF, o que pode explicar maior reconhecimento do mecanismo nesse nível. Entretanto, não se avançou em relação aos prontuários compartilhados, sistema de informação vertical^12^, presentes em apenas 14% das equipes de atenção básica no país, sobretudo em municípios com mais de 100 mil habitantes^22^. Por não serem compartilhados, em contexto de provedores pequenos e independentes, os profissionais não conseguem acessar os registros clínicos nos diversos serviços da RAS^20^.

Entre os mecanismos de coordenação por padronização^12^, protocolos compartilhados e sessões clínicas eram ainda mais residuais, principalmente na AE. Estudo realizado em duas RAS de Pernambuco também revelou baixo conhecimento por médicos da AE acerca dos protocolos elaborados pelo Ministério da Saúde^4^, o que parece colocar sob interrogação seu caráter “compartilhado”. Embora menos conhecidos que os instrumentos de *feedback*, havia alta utilização, mais expressiva na APS, ou seja, aqueles que conheciam utilizavam os protocolos para padronizar a atenção, como encontrado por Oliveira et al.^4^ Ao mesmo tempo, os profissionais apontaram dificuldades para incorporar as diretrizes protocolares, pois a RAS não ofertava os insumos e serviços necessários. Já as sessões clínicas não faziam parte do processo de trabalho da AE, resultado também encontrado em outro estudo^4^, e estiveram parcialmente presentes na APS, sendo necessária garantia de tempo, por parte da gestão, para viabilizar a adesão a esse mecanismo de coordenação^9^.

Este estudo não analisou possíveis associações entre conhecimento e uso dos mecanismos de coordenação pelos médicos, assim como não abrangeu outros atores e fontes de informação que pudessem precisar sua implementação e utilização na RAS, como monitoramento das referências, frequência de realização de sessões clínicas e disponibilização de protocolos, entre outras. Destaca-se que o estudo utilizou um instrumento aplicado em cenários nacional e internacional, que se mostrou adequado à identificação e análise em profundidade de mecanismos de coordenação, caracterizando e qualificando aspectos do uso que podem ser apropriados por pesquisadores e gestores da saúde implicados na melhoria da coordenação do cuidado.

## CONCLUSÕES

O conjunto de atribuições da APS vem aumentando ao longo do tempo, com sucessiva incorporação de atenções antes prestadas em outros níveis^20^ e, no caso brasileiro, descentralização de ações e programas^23^, além de aumento da pressão assistencial, atribuída, entre outros fatores, à insuficiência de recursos humanos^9^ e excesso de usuários por equipe^14^. Problemas relacionados à insuficiente oferta de serviços na RAS^14^ também desestimulam e dificultam o uso de mecanismos de padronização. Nesse cenário, parece consensual que atividades de coordenação não surgirão espontaneamente^20^, sem garantia de condições de trabalho adequadas como tempo suficiente, pagamento por performance para atividades de coordenação^20^ e vínculos trabalhistas estáveis que permitam aos profissionais desenvolvê-las, assim como processos formativos voltados ao trabalho em rede e reconhecimento do papel da APS^8^^,^^9^.

No caso do cuidado em saúde, a formação de redes e produção de conexão entre trabalhadores é um imperativo, visto não haver autossuficiência^24^. O envolvimento do conjunto dos trabalhadores do SUS, inclusive nos processos investigativos, parece ser o caminho mais promissor e possível, na medida em que mecanismos de ajustamento mútuo facilitam a aproximação e a comunicação direta entre profissionais bem como contribuem para aumentar a confiança interprofissional^12^. Em alguns casos, tais mecanismos são caracterizados como tecnologias leves, que poderiam ser incentivadas sem necessidade de grande aporte de recursos.

Por fim, argumenta-se que mecanismos de coordenação, no nível das práticas, apresentam grande potencial para melhorar a colaboração interprofissional interníveis^2^. Em sentido complementar, tão importante quanto implementar aqueles que ainda não existem (como prontuários eletrônicos compartilhados) é criar condições adequadas para a utilização de tais mecanismos, muitos dos quais, conforme mostrou este estudo, já estão presentes na RAS. É na complementariedade de medidas que possibilitem conhecer, desenvolver habilidades profissionais, institucionalizar e promover condições organizacionais para a efetiva utilização de mecanismos de coordenação em toda RAS que a fragmentação do sistema e dos cuidados poderá ser enfrentada.

## Footnotes

a Samico I, Albuquerque PC, Vázquez ML, Vargas I, Unger JP, De Paepe P, et al. Questionário de articulação/coordenação entre os níveis de atenção: COORDENAR-BR. Recife (PE): Instituto Medicina de Integral Prof. Fernando Figueira (IMIP); 2015 [citado 9 jul 2018]. Disponível em: http://www.equity-la.eu/upload/seccions/files/COORDENA_BR%282%29.pdf
